# Uterine Lavage Identifies Cancer Mutations and Increased *TP53* Somatic Mutation Burden in Individuals with Ovarian Cancer

**DOI:** 10.1158/2767-9764.CRC-22-0314

**Published:** 2022-10-27

**Authors:** Talayeh S. Ghezelayagh, Brendan F. Kohrn, Jeanne Fredrickson, Enna Manhardt, Marc R. Radke, Ronit Katz, Heidi J. Gray, Renata R. Urban, Kathryn P. Pennington, John B. Liao, Kemi M. Doll, Elise J. Simons, Jennifer K. Burzawa, Barbara A. Goff, Paul Speiser, Elizabeth M. Swisher, Barbara M. Norquist, Rosa Ana Risques

**Affiliations:** 1 Department of Obstetrics and Gynecology, University of Washington, Seattle, Washington.; 2 Department of Laboratory Medicine and Pathology, University of Washington, Seattle, Washington.; 3 Medical University of Vienna, Vienna, Austria.

## Abstract

Current screening methods for ovarian cancer have failed to demonstrate a significant reduction in mortality. Uterine lavage combined with *TP53* ultradeep sequencing for the detection of disseminated ovarian cancer cells has emerged as a promising tool, but this approach has not been tested for early-stage disease or non-serous histologies. In addition, lavages carry multiple background mutations, the significance of which is poorly understood. Uterine lavage was collected preoperatively in 34 patients undergoing surgery for suspected ovarian malignancy including 14 patients with benign disease and 20 patients with ovarian cancer [6 non-serous and 14 high-grade serous-like (serous)]. Ultradeep duplex sequencing (∼3,000×) with a panel of common ovarian cancer genes identified the tumor mutation in 33% of non-serous (all early stage) and 79% of serous cancers (including four early stage). In addition, all lavages carried multiple somatic mutations (average of 25 mutations per lavage), more than half of which corresponded to common cancer driver mutations. Driver mutations in *KRAS, PIK3CA, PTEN, PPP2R1A*, and *ARID1A* presented as larger clones than non-driver mutations and with similar frequency in lavages from patients with and without ovarian cancer, indicating prevalent somatic evolution in all patients. Driver *TP53* mutations, however, presented as significantly larger clones and with higher frequency in lavages from individuals with ovarian cancer, suggesting that *TP53*-specific clonal expansions are linked to ovarian cancer development. Our results demonstrate that lavages capture cancer cells, even from early-stage cancers, as well as other clonal expansions and support further exploration of *TP53* mutation burden as a potential ovarian cancer risk factor.

**Significance:**

Cancer driver mutations are found in uterine lavage DNA in all individuals, but driver *TP53* mutations presented as significantly larger clones and with higher frequency in lavages from individuals with ovarian cancer. This suggests that *TP53*-specific clonal expansion plays a role in tumorigenesis and presents opportunities for early detection.

## Introduction

Ovarian cancer is a prominent cause of cancer-related mortality, with more than 200,000 annual deaths worldwide (1, 2). Because of the indistinct symptoms experienced during disease progression, ovarian cancer is most often diagnosed at an advanced stage, unfortunately leading to high mortality rates. Five-year survival for advanced stage disease is still only 30%, while survival for early stage is >90% (3). This has prompted efforts to develop screening and early detection methods, including transvaginal ultrasound and cancer antigen 125 (CA-125) serum testing (4, 5), but none has thus far demonstrated a significant reduction in mortality (6). Explorations into liquid biopsies using pap tests have shown limited sensitivity for ovarian cancer detection (7–9).

Lavage of the uterine cavity with 10 mL of saline using a three-way catheter can detect exfoliated cancer cells from the ovary or fallopian tube (10, 11) and be safely performed in an outpatient setting (12), suggesting a novel noninvasive screening tool. Using next-generation sequencing (NGS) and droplet digital PCR to identify the known tumor mutation, Maritschnegg and colleagues demonstrated that 80% of uterine lavages from individuals with ovarian cancer carried tumor DNA (10). Our group then performed blinded uterine lavage analysis using duplex sequencing (DS), an ultra-accurate ultrasensitive NGS approach (13, 14), and demonstrated detection of cancer-specific *TP53* mutations in 80% of samples from high-grade serous cancer (HGSC) cases (11). An advantage of DS is that it allows for the detection of not only tumor DNA but also low-frequency mutant clones (9, 11, 15, 16), which are now recognized as the result of prevalent somatic clonal evolution (17, 18) and might be linked to cancer development (19, 20). Supporting this notion, we have shown that Pap test DNA carries more non-tumor *TP53* pathogenic mutant clones in individuals with ovarian cancer (9), but this has not been properly tested in lavage DNA. In addition, the combined approach of uterine lavage plus DS has not been tested for non-serous ovarian cancer or early-stage disease, which are important aspects for widespread implementation of a clinical test for early ovarian cancer detection.

While 75% of epithelial ovarian cancer cases have high-grade serous histology, the remaining 25% include low-grade serous, clear cell, endometrioid, and mucinous histologies. Non-serous carcinomas are typically driven by a variety of genetic alterations including activation of *PIK3CA* and the Wnt-β-catenin pathway, and inactivation of *ARID1A* and *PTEN* (21). Furthermore, while the majority of HGSC harbor a *TP53* mutation, up to 20% of cases may not (22–25). Thus, any ovarian cancer screening method utilizing detection of cancer-driving mutations must expand beyond *TP53* to improve sensitivity. In this study, we aimed to pilot the combination of uterine lavage with ultradeep sequencing using an expanded gene panel to improve detection of both early-stage and non-serous ovarian cancer. In addition, we aimed to leverage the extreme sensitivity of ultradeep DS to characterize background somatic mutations in lavages and determine potential associations with ovarian cancer.

## Materials and Methods

### Patients and Samples

The study included 34 patients who underwent surgery with preoperative concern for an ovarian malignancy at the University of Washington (Seattle, WA) between November 2019 and October 2020. Inclusion criteria included the presence of a uterus, and at least one ovary and fallopian tube. Patients undergoing neoadjuvant chemotherapy before surgery were excluded because of the potential impact on sequencing findings. The study was designed in accordance with recognized ethical guidelines for patient participation. Patients were enrolled prior to surgery under an Institutional Review Board–approved protocol at the University of Washington (Seattle, WA) and provided informed written consent for tissue collection, including tumor and a preoperative uterine lavage. Uterine lavages were collected after induction of anesthesia and vaginal antiseptic preparation using a transcervical catheter (Ovartec) as described previously (10, 12). The clinicopathological characteristics of the patients are included in Supplementary Table S1 and further described in Supplementary Materials and Methods. In most cases, carcinoma was detected on intraoperative pathology and a staging procedure was performed per the surgeon's usual practice. One case of stage IB HGSC was not staged as intraoperative pathology was benign. Patient sample numbering for the article was assigned on the basis of histology and age. During this time period, which included the first year of the SARs-Co-V2 pandemic, many patients with advanced ovarian cancer received neoadjuvant chemotherapy to reduce perioperative morbidity and mortality, and were thus excluded from this study. This led to a higher proportion of early-stage cancers in this sampled population.

Samples were stored at the University of Washington Gynecologic Oncology Tissue Bank. Lavages were mixed with an ethanol-based stabilization medium and filtered with a gravity flow 100 μm cell strainer to remove potential clusters of endometrial cells. Filtered samples were then centrifuged at 300 × *g* for 10 minutes and cell pellets were frozen at −80°C. A subset of samples underwent additional centrifugation to increase size of cell pellet. Genomic DNA was extracted from cell pellets using the Dneasy Blood & Tissue Kit (Qiagen) with proteinase K digestion at 37°C for 2 hours and including RNAse treatment. DNA was quantified by Qubit dsDNA BR Assay Kit (Thermo Fisher Scientific) and stored at −80°C until library preparation. Sartorius Vivacon 500 DNA concentrators were used if needed prior to library preparation.

### Lavage DNA Sequencing

A total of 200 ng of lavage DNA were processed for DS using commercially available kits (TwinStrand Biosciences). Library preparation consisted of sonication, end-repair, A-tailing, ligation to duplex adapters, fragment amplification, hybridization capture with 120 bp biotinylated probes (*TP53* human panel v1.0 from TwinStrand Biosciences, and xGen Hyb probes from Integrated DNA Technology, for the rest of genes), and library amplification. The capture panel was designed to target candidate ovarian cancer driver genes previously identified as the most common drivers in endometrial, clear-cell and high-grade serous carcinomas according to the Catalogue of Somatic Mutations in Cancer (COSMIC; v95, accessed on January 24, 2022; ref. 26). The panel included *TP53*, *ARID1A*, *PTEN*, *PPP2R1A*, *CDKN2A*, *KRAS* (whole genes; Supplementary Table S2); and *CTNNB1*, *PIK3CA*, and *BRAF* (hotspots only; Supplementary Table S3). The total size of the coding region captured was 12.1 Kb. Given the small size of the panel, two rounds of hybridization capture were performed to increase efficiency (27). Proper library fragment size was confirmed by Agilent 4200 TapeStation. Libraries were quantified using the Qubit dsDNA HS Assay kit, diluted, and pooled for sequencing. Libraries were sequenced using 150 PE reads on a HiSeq at Genewiz, allocating approximately 13 million clusters per sample.

### Data Analysis

Sequencing reads were analyzed as described previously (13) using pipeline v2.1.2 from https://github.com/Kennedy-Lab-UW/Duplex-Seq-Pipeline. Variant Call Format (VCF) files were converted to MAF files, which were then postprocessed with R version 4.1.2 (ref. 28; Supplementary Materials and Methods). For each sample, the overall duplex depth per gene was calculated as the average depth of all on-target coding nucleotides sequenced (Supplementary Table S4). For each mutation, variant allele frequency (VAF) was calculated as the number of mutant duplex reads divided by the total duplex depth at the given position. To correct for the variability in sequencing depth across samples, sample comparisons were made based on mutation frequency (MF) and mutation burden (MB), calculated for each gene and overall for all the genes in the study. MF was calculated as the number of mutant positions divided by the total number of duplex nucleotides sequenced, and MB was calculated as the number of total mutant duplex reads (each mutant duplex read corresponds to a single DNA mutant molecule) divided by the total number of duplex nucleotides sequenced (Supplementary Table S4).

COSMIC data (26) were used to determine the codon location of substitutions reported for ovarian carcinomas for the genes and transcripts of interest. The histograms of mutation location for each gene were then compared with the histograms obtained with mutations identified in uterine lavage. In addition, COSMIC data were used to determine ovarian cancer hotspot codons, which were defined as codons with two or more substitutions and accounting for at least 1% of the reported substitutions for ovarian carcinoma in each gene (Supplementary Table S5). For oncogenes in the study (*BRAF*, *CTNNB1*, *KRAS*, and *PIK3CA*), cancer driver mutations were defined as missense mutations in ovarian cancer hotspot codons. For tumor suppressor genes (*CDKN2A*, *PTEN*, *PPP2R1A*, *ARID1A*, and *TP53*), cancer driver mutations were defined as missense mutations in ovarian cancer hotspot codons plus any nonsense, insertion/deletion (indel) or splice mutations. The list of annotated coding mutations is shown in Supplementary Table S6.

### Tumor Sequencing

Tumor DNA was sequenced to identify driver mutations and compare with mutations found in the lavages. For five of the 13 high-grade serous cases in the study, the *TP53* tumor driver mutation had been previously identified using the targeted BROCA panel as part of a larger institutional study (Table 1; refs. 29, 30). For the remaining eight high-grade serous tumors and all the non-serous tumors, DNA was extracted from formalin-fixed, paraffin-embedded tissue sections and duplex sequenced using a MiSeq Illumina platform on site as detailed in Supplementary Materials and Methods.

**TABLE 1:** Tumor and uterine lavage mutation testing

					Tumor testing				Uterine lavage testing			
Patient ID	Ovarian cancer type	Histology	Age at surgery	Stage	Tumor testing platform	Mutation	Protein variant	Coding variant	Tumor mutation found	VAF	Mutant duplex reads	Duplex depth at position
P15	Non-serous	Clear-cell carcinoma	53	IC3	DS	ARID1A	p.W1023Vfs*10	c.3066dup	yes	0.0134	14	1046
P16	Non-serous	Clear-cell carcinoma	64	IIB	DS	TP53	P.E221*	c.661G>T	no			
P17	Non-serous	Clear-cell carcinoma	69	IA	DS	ARID1A	p.K1808Nfs*4	c.5423 5424insTTAC	no			
P18	Non-serous	Endometrioid carcinoma	57	IC1	DS	CTNNB1	P.S33C	c.98C>G	no			
P19^a^	Non-serous	Endometrioid carcinoma	58	IC3	DS	ARID1A CTNNB1 PIK3CA	p.R1335* p.D32Y P.H1047R	C.40030T c.94G>T c.3140A>G	no			
P20	Non-serous	Endometrioid carcinoma	83	IA	DS	PIK3CA	P.N1044Y	c.3130A>T	yes	0.0053	16	2997
P21	Serous	Carcinosarcoma	51	IB	DS	TP53	p.X307 splice	c.919+1G>A	yes	0.0075	42	5593
P22	Serous	HGSC	52	IIIC	BROCA	TP53	P.G244V	c.731G>T	yes	0.0050	21	4166
P23	Serous	HGSC	56	IIB	BROCA	TP53	p.R342Tfs*4	c.1023 1024insAC	yes	0.0005	2	3835
P24	Serous	HGSC	57	IB	DS	TP53	P.C275F	c.824G>T	yes	0.0169	97	5723
P25	Serous	HGSC	61	IIA	DS	TP53	P.V157F	C.469G>T	no			
P26	Serous	HGSC	62	IIIB	DS	TP53	P.R273C	c.817C>T	yes	0.0037	22	5905
P27	Serous	HGSC	67	IIIA1	BROCA	TP53	p.V274A	c.821T>C	yes	0.0005	2	3938
P28	Serous	HGSC	68	IVB	DS	TP53	P.V143M	c.427G>A	no			
P29	Serous	HGSC	68	IVB	DS	TP53	p.S315Rfs*26	c.942 954del	no			
P30	Serous	HGSC	71	IIIC	DS	TP53	P.H193Y	c.577C>T	yes	0.0091	56	6130
P31	Serous	HGSC	72	IIIC	BROCA	TP53	P.S241F	C.722OT	yes	0.0045	9	1999
P32	Serous	HGSC	73	IIIC	DS	TP53	p.X126 splice	c.376-2A>G	yes	0.0002	1	4291
P33	Serous	HGSC	76	IIA	BROCA	TP53	P.P151H	c.452C>A	yes	0.0962	372	3866
P34	Serous	HGSC	83	IIB	DS	TP53	p.S261Vfs*84	c.780del	yes	0.1832	926	5055

Abbreviation: DS, duplex sequencing; HGSC, high-grade serous carcinoma.

^a^Note that patient P19 had three tumor driver mutations.

### Statistical Analysis

Comparison of MF, MB, and VAF across groups of individuals was performed by Mann–Whitney *U* test. Correlations were tested with Spearman rank test. Associations between categorical variables were tested with Fisher exact test. Two logistic regression models were constructed, one including the standard ovarian cancer risk variables (age and CA-125) and an exploratory model including age, CA-125, and *TP53* MB. The models equated the relationship between variables with the occurrence of ovarian cancer to estimate beta coefficients with 95% confidence intervals. Because of a heavy right-tailed distribution, CA-125 was log transformed. Age and *TP53* MB were presented as a per SD increase. All tests were two sided at an alpha level (type 1 error rate) of 0.05. Statistical analyses were performed with SPSS version 26 (31), R version 4.1.1 (28), and Stata 16 (32).

### Software Availability

The Duplex Sequencing Pipeline is available at https://github.com/Kennedy-Lab-UW/Duplex-Seq-Pipeline.

### Data Availability Statement

Sequencing data from this study are available at the NCBI BioProject database (https://www.ncbi.nlm.nih.gov/bioproject) under BioProject ID PRJNA879769.

## Results

Uterine lavage was collected preoperatively from 34 patients that underwent gynecologic surgery for suspected ovarian cancer using commercial catheters (Supplementary Fig. S1A; Supplementary Table S1). Cell pellet DNA was analyzed with ultradeep DS using a panel of genes frequently mutated in the most common ovarian cancer histologies (Supplementary Fig. S1B; Supplementary Tables S2 and S3). Average depth of sequencing in target coding regions was 3,222x (minimum 1,203x, maximum 4,164x). Routine pathologic review of surgical specimens revealed that 14 patients had benign masses and 20 patients had ovarian cancer, including clear-cell carcinoma (3), endometrioid carcinoma (3), carcinosarcoma (1), and HGSC (13). The carcinosarcoma case had a significant serous component and was combined with the HGSC cases in a “serous” histology group. A total of 12 of the 20 patients with ovarian cancer (60%) had early-stage disease (FIGO stage I or II), providing a unique opportunity to test the sensitivity of this approach for early ovarian cancer detection (Supplementary Fig. S1C). One patient had prior exposure to chemotherapy for breast cancer and two patients with HGSC were later identified to carry germline *BRCA2* mutations (Supplementary Table S1).

### Uterine Lavage Detected the Tumor Mutation in More Than Two-thirds of Ovarian Cancer Cases

For the 20 patients with ovarian cancer, neoplastic DNA was sequenced to determine whether the ovarian cancer mutation was present in the lavage. Non-serous cancers had mutations in a variety of genes including *ARID1A, CTNNB1, PIK3CA,* and *TP53*, whereas the serous cancers were driven exclusively by *TP53* mutations (Table 1). In total, 13 of 20 (65%) of the tumor mutations were identified in the corresponding lavage, but the rate of detection was higher in serous ovarian cancer (11/14, 79%) than in non-serous cancers (2/6, 33%). Because duplex reads correspond to unique DNA molecules, the VAF of mutations is a direct readout of clone size. The tumor clones identified in lavage ranged from 0.02% to 18% of the sequenced DNA. The VAF of the tumor mutation in lavage was not associated with tumor stage or blood levels of antigen CA-125 (Supplementary Fig. S2).

**FIGURE 1:**
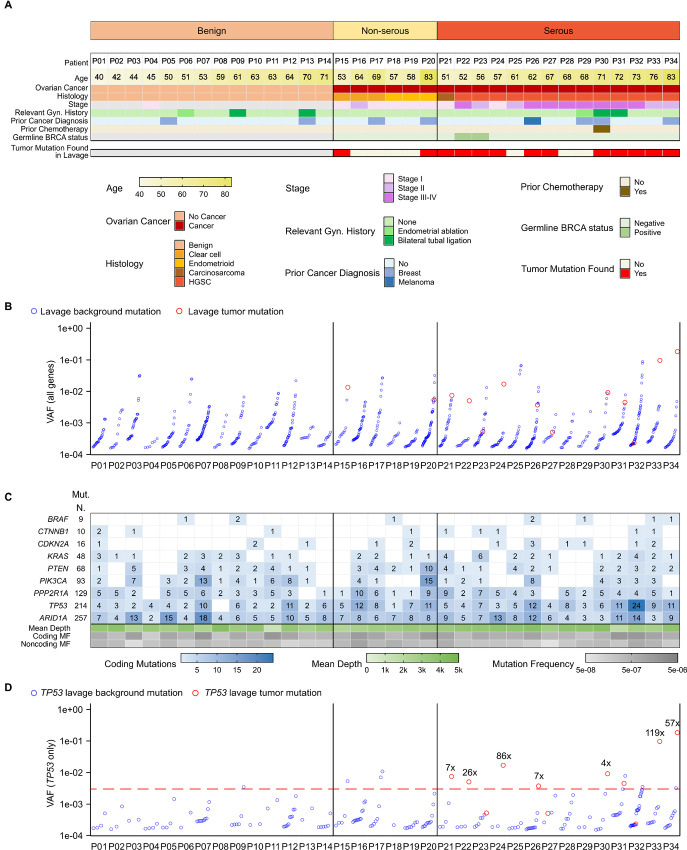
Summary of mutations identified in lavages from patients with and without ovarian cancer. **A,** Clinicopathological characteristics of the patients are color coded according to the legend. Patients are sorted by ascending age within each histologic group. For patients with ovarian cancer, it is indicated whether the tumor mutation was found in the uterine lavage. **B,** The VAF for all mutations detected in uterine lavage samples is displayed. Red circles correspond to tumor mutations and blue circles correspond to other background mutations, sorted by ascending VAF within each patient. **C,** The total number of mutations identified for each gene in lavage samples is indicated (Mut. N) as well as the number of mutations in each lavage (blue gradient boxes). The mean depth of sequencing for coding exons is indicated with a gradient scale in green. Coding and noncoding MF, corresponding to the count of unique mutations adjusted by nucleotides sequenced, are indicated with a gradient scale in gray. **D,** The VAF for all *TP53* mutations detected in each uterine lavage sample is displayed, with the red circles indicating tumor mutations. The ratio of the VAF of the tumor mutation to the VAF of the largest background mutation is indicated if >4x. Red line indicates a potential VAF cutoff of 0.003.

We next explored whether the identification of the tumor mutation in uterine lavage was related to clinicopathological characteristics (Fig. 1A). Of seven lavages in which the tumor mutation was not identified, four were in early-stage non-serous cancers, and three were in serous cancers. One of the serous cancers was early stage and another corresponded to an individual with a prior endometrial ablation. Remarkably, the tumor mutation was identified in five of six (83%) lavages from stage I–II serous ovarian cancer, indicating that early stage was not a factor preventing detection and suggesting early transit of cancerous clones. In addition, the tumor clone was also detected in the lavages of two patients that were in their 70s and had undergone a prior bilateral tubal ligation, which was unexpected. One patient had fallopian tube involvement, which may have locally spread to the proximal tubal fragment, though this could not be confirmed. The other patient had uterine myometrial involvement of tumor. Alternatively, cells carrying these mutations might have traveled not through the tubal lumen but via lymphatic or hematologic channels, or these clones may represent nonmalignant parallel somatic evolution.

In addition to tumor mutations, lavages carried other background mutations (Fig. 1B; Supplementary Table S6). These mutations were identified in all the genes sequenced and in patients with and without ovarian cancer (Fig. 1C). In a subset of patients with ovarian cancer, the tumor mutation had a VAF higher than background mutations (Fig. 1B), indicating that the tumor clone was the largest in the lavage. In lavages from other patients with ovarian cancer, however, background mutations obscured the tumor mutation. We observed that many of the large background clones corresponded to genes other than *TP53*, which are relevant for non-serous histologies but generally not for serous ovarian cancer (Supplementary Fig. S3). Thus, we restricted the analysis to *TP53* to determine whether focusing on this main driver gene could help distinguish tumor mutations from background (Fig. 1D). In seven of 11 lavages from serous ovarian cancer cases in which the tumor mutation was identified (63%), the mutation was present with VAF greater than 0.003 and more than 4-fold higher VAF than the largest background mutation. Comparatively, only 1 of 14 patients without cancer had a *TP53* mutation VAF greater than 0.003. These results demonstrate clear identification of *TP53*-mutant tumor DNA in a large fraction of lavages despite background *TP53* mutations.

### Lavages Had an Excess of Coding versus Noncoding Background Mutations and Showed Higher Frequency of *TP53* Mutations in Patients with Ovarian Cancer

We next explored whether background mutations harbored relevant biological information that could help distinguish patients with and without ovarian cancer. All lavages carried multiple mutations in at least two or more genes (Fig. 1C) with an average of 25 mutations per lavage (min = 6, max = 54). The genes with the least mutations were *BRAF* and *CTNNB1* and the ones with the most mutations were *TP53* and *ARID1A,* although these differences were partially due to variation in the size and depth of the regions sequenced for each gene. In general, lavages that showed large numbers of mutations in one gene also showed large numbers of mutations in other genes. Gene-specific MF were calculated to adjust for depth of sequencing (Supplementary Table S4). The MF of all the genes were highly correlated (Supplementary Fig. S4) confirming that samples with high levels of background mutations carried them across multiple genes.

**FIGURE 2:**
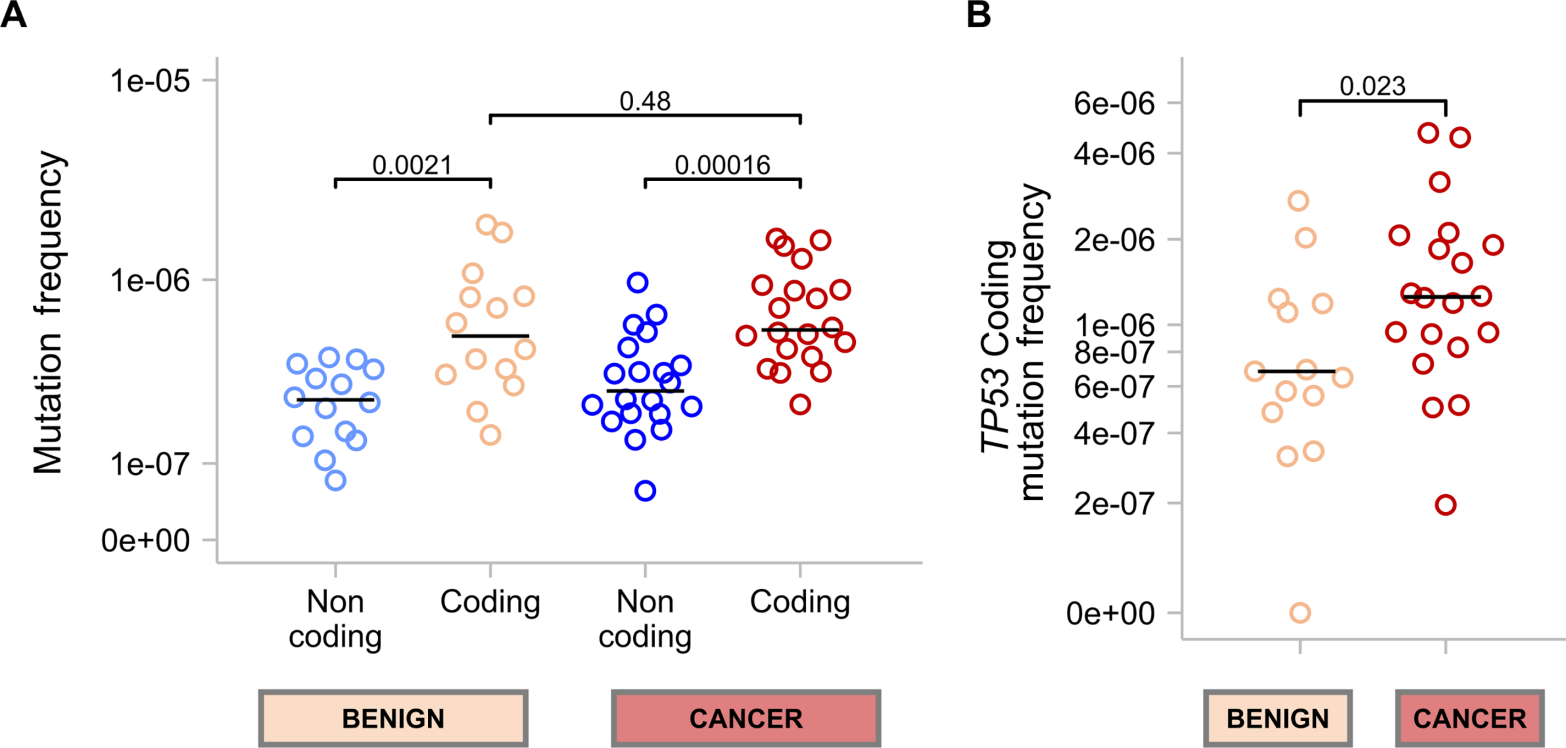
Comparison of MF in patients with and without ovarian cancer. MF is calculated by dividing the number of mutations detected by the number of nucleotides sequenced. Each circle represents the MF for an individual sample. *P* values for Mann–Whitney *U* tests are shown for each comparison. The median MF for each group is indicated with a horizontal black bar. **A,** Comparison of coding and noncoding MF for all sequenced genes combined in uterine lavages from benign and cancer cases. **B,** Comparison of *TP53*-specific coding MF between benign and cancer cases.

We then determined the overall coding MF (all genes) and noncoding MF because the target panel also captured intronic regions that contained mutations in all samples. Coding MF was significantly higher than noncoding MF in lavages from patients with and without ovarian cancer (Fig. 2A). When ovarian cancer cases were separated into non-serous and serous, the difference remained significant for serous ovarian cancer (Supplementary Fig. S5A). While noncoding mutations reflect mutagenic processes, the excess of coding mutations in lavage suggests clonal expansions of cells with functional mutations, as described previously (11, 19). The overall coding MF was not significantly different between patients with and without ovarian cancer, indicating that clonal expansions in the selected genes, as a whole, occur similarly in both groups. However, when comparing coding MF by gene, we observed that background mutations in *TP53* were more abundant in lavages of patients with ovarian cancer than those without cancer (Fig. 2B). When separating cancer cases into serous and non-serous, there were not significant differences in lavage *TP53* MF between the two groups (Supplementary Fig. S5B). Both cancer groups, however, had increased *TP53* MF compared with the benign group although the difference was significant only for serous cases (Supplementary Fig. S5B). For the rest of the genes in the study, there were not significant differences between the MF in lavages from cancer and benign cases (Supplementary Fig. S6). *TP53* was also the only gene whose coding MF was positively correlated with age (Supplementary Fig. S7A), consistent with prior studies (9, 11, 15). This association, however, was influenced by the fact that the oldest patients in the study had ovarian cancer and high *TP53* MF (Supplementary Fig. S7B).

### More Than Half of the Mutations in Uterine Lavage are Common Ovarian Cancer Driver Mutations, Many of Which are Expanded in Larger Clones Compared to Non-driver Mutations

**FIGURE 3:**
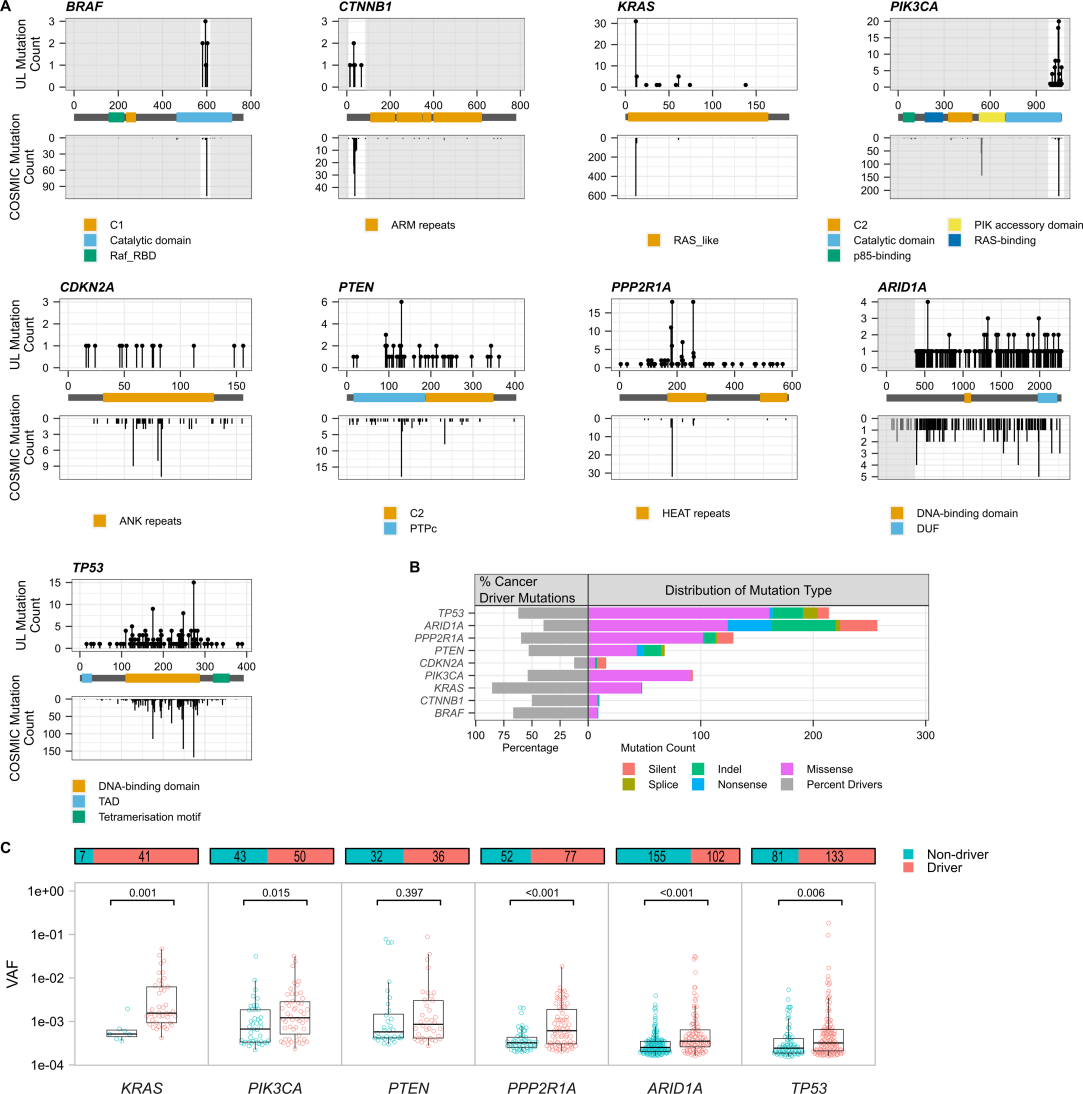
Characterization of uterine lavage mutations by gene. **A,** The location and distribution of mutations in uterine lavage samples mirror those identified in ovarian carcinomas. Top panels show the location by codon position of somatic mutations identified in uterine lavage, with mutation counts plotted on the *Y* axis. Lower panels show mutations identified in ovarian carcinomas in COSMIC. Indels are excluded as they might span multiple codon locations. Areas of the gene not captured in the DS panel are grayed out. Gene domains are highlighted in the legends. **B,** Percentage of cancer driver mutations identified for each gene in the entire cohort (left) and distribution of mutation type per gene (right). Cancer driver mutations are defined, in oncogenes, as substitutions occurring in common hotspots codons and, in tumor suppressor genes, as substitutions occurring in common hotspots codons plus insertion/deletions, nonsense, and splice mutations. **C,** The VAF of driver versus non-driver mutations identified in the entire cohort is displayed by gene. Only genes that exhibited mutations in >50% of the uterine lavage samples are shown. Each circle represents a unique mutation. Overlying box plots display the quartiles with whiskers extending up to 1.5× the interquartile range. Bar plots above display the total number of mutations in each group and the distribution between driver and non-driver. *P* values correspond to Mann–Whitney *U* tests.

To gain further insight into the nature of lavage mutations, we plotted them along the coding region of each gene and compared their distribution with the distributions obtained for ovarian cancer mutations reported in COSMIC (Fig. 3A). Overall, the distribution of mutations was very similar in lavages and in COSMIC, indicating that lavage mutations are not random, but mimic mutations found in ovarian cancer, even in the absence of ovarian cancer. One remarkable exception was *BRAF* p.V600E, which accounts for 78.8% of *BRAF* mutations in ovarian cancer but was not observed in lavages. Lavages, however, carried mutations in other *BRAF* hotspot codons common in ovarian cancer albeit at much lower frequency (codons 594, 581, and 597, representing 2.9%, 2.2%, and 2.2% of BRAF mutations, respectively).

On the basis of ovarian cancer data from COSMIC, we then determined what proportion of the mutations identified in lavages could be considered cancer driver mutations. Of 844 coding mutations identified in lavages, more than half (452, 54%) qualified as cancer driver mutations (Supplementary Table S6). With the exception of *CDKN2A*, which carried few mutations overall, all genes carried high levels of cancer driver mutations ranging from 40% in *ARID1A* to 85% in *KRAS* (Fig. 3B). While these proportions showed some variation across samples, especially for tumor suppressor genes (Supplementary Fig. S8A), they were not significantly different between lavages from patients with and without ovarian cancer (Supplementary Fig. S8B) indicating that clonal expansions of driver mutations in lavage are prevalent irrespective of ovarian cancer progression.

The types of mutations observed for each gene corresponded to expectations based on their roles as oncogenes or tumor suppressor genes. Oncogenes (*PIK3CA*, *KRAS*, *CTNNB1,* and *BRAF*) carried mostly missense mutations whereas tumor suppressor genes (*TP53*, *ARID1A*, P*PP2R1A*, *PTEN,* and *CDKN2A*) were enriched for indels, nonsense and splice mutations (Fig. 3B). The analysis of the overall mutational spectrum also demonstrated a high resemblance between lavage mutations and mutations observed in ovarian cancers (Supplementary Fig. S9). The spectrum was characterized by an enrichment of C>T mutations in lavages from patients with non-serous and serous ovarian cancer as well as lavages from patients older than 50 years of age, consistent with the pattern observed in ovarian cancer and the age-related origin of C>T mutations (33).

We hypothesized that cells carrying cancer driver mutations might be more likely to clonally expand than cells without driver mutations, resulting in overall higher VAF for driver mutations. To test this hypothesis, for the six genes that exhibited mutations in >50% of the uterine lavage samples, we compared the VAF of non-driver versus driver mutations (Fig. 3C). Despite most mutations being present at very low VAF (<0.01), we observed that for all the genes except *PTEN*, the VAF of driver mutations was significantly higher than the VAF of non-driver mutations. These results demonstrate that lavage DNA carries large clones driven by common cancer driver mutations and that ultradeep sequencing can quantify their size, and thus demonstrate their expansion, compared with non-driver mutations.

### *TP53* MB in Uterine Lavage is Higher in Patients with Ovarian Cancer and has Significant Predictive Value Over Age and CA-125

**FIGURE 4:**
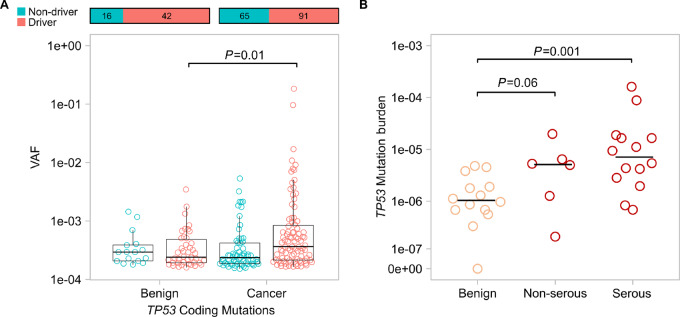
Uterine lavages from patients with ovarian cancer carry more large clones with *TP53* driver mutations and show higher *TP53* mutation burden than lavages from patients without cancer. **A,** Comparison of VAF of *TP53* driver mutations in lavage DNA from patients with and without ovarian cancer. Cancer driver mutations in *TP53* include substitutions occurring in common hotspots codons plus insertion/deletions, nonsense, and splice mutations. Each circle corresponds to a unique mutation. Overlying box plot displays the quartiles with whiskers extending up to 1.5× the interquartile range. Bar plots above display the total number of mutations in each group and the distribution between driver and non-driver. *P* values correspond to Mann–Whitney *U* test comparing the distribution of VAF of cancer driver mutations between patients with benign and cancer. **B,***TP53* mutation burden, calculated as the total number of *TP53-*mutant molecules identified in a lavage divided by the total number of nucleotides sequenced, is compared between patients with benign disease, non-serous ovarian cancer, and serous ovarian cancer (high-grade serous and carcinosarcoma). Each circle corresponds to an individual uterine lavage sample. Horizontal bars indicate the median for each group and *P* values correspond to Mann–Whitney *U* tests.

We then wondered whether the size of clones driven by cancer driver mutations was larger in patients with ovarian cancer than in those without. For all the genes with mutations in more than 50% of lavages, we plotted the VAF of cancer driver mutations in patients with and without ovarian cancer. Interestingly, we did not observe significant differences in any of the genes (Supplementary Fig. S10), with the exception of *TP53* (Fig. 4A). Patients with and without ovarian cancer had similar proportions of cancer driver mutations, but the VAF of *TP53* driver mutations was significantly higher in those with ovarian cancer, indicating larger clonal expansions.

Given that ovarian cancer was associated with the number of *TP53* mutations as well as the size of mutant clones, we reasoned that *TP53* MB should be the best metric to discriminate patients with and without ovarian cancer because it counts not only the number of mutated positions, but the number of mutant molecules in each position, reflecting both number and size of clones. Thus, for all the lavages in the study, we calculated the MB of each gene (Supplementary Fig. S11). As expected, we did not observe significant differences in the MB of cancer and benign lavages except for *TP53*. Patients with ovarian cancer had significantly higher *TP53* MB than those without ovarian cancer (*P* = 0.001). Notably, when separating by histology, we observed that *TP53* mutation burden tended to be higher in serous as well as non-serous ovarian cancer (Fig. 4B). These results suggest that *TP53* clonal evolution might be related not only to the development of HGSC, but also non-serous cancer despite not being the most common driver of those cancer types.

As with *TP53* MF, *TP53* MB was also significantly associated with age (Spearman correlation, *P* = 0.003). Because samples were consecutively collected and not age matched, we performed a sensitivity study restricting the comparison of *TP53* MB with patients with and without cancer in the same age range (>50 and <72). *TP53* MB was higher in patients with ovarian cancer with borderline significance (*P* = 0.055).

Finally, to test whether *TP53* MB could provide clinical value for the detection of ovarian cancer in patients with pelvic masses, we built two logistic regression models to compare the predictive value of CA-125 and age versus CA-125, age, and *TP53* MB (Table 2). We found that *TP53* MB was significant (*P* = 0.036) even when accounting for CA-125 and age, suggesting potential value of *TP53* MB to improve the predictive value of the current markers. This significant association was retained even when restricting to serous ovarian cancer, despite the smaller sample size. Given the strong associations observed for *TP53* MB and ovarian cancer despite low numbers, future larger studies are warranted to confirm these findings.

**TABLE 2:** Logistic regression for ovarian cancer prediction

	All patients (*n* = 34)		Non-serous cancer excluded (*n* = 28)	
	β (95% Cl)	*P*	β (95% Cl)	*P*
**Current predictive variables**				
Age (per SD increase)^a^	1.08 (0.01–2.15)	0.048	1.16 (−0.05 to 2.37)	0.061
CA-125 (log tranformed)	0.66 (0.09–1.23)	0.024	0.73 (0.10–1.35)	0.023
**Exploratory model**				
*TP53* mutation burden (per SD increase)^b^	19.14 (1.29–36.98)	0.036	24.82 (0.39–49.26)	0.046
Age (per SD increase)^a^	1.15 (−0.63 to 2.92)	0.205	2.05 (−0.98 to 5.07)	0.185
CA-125 (log tranformed)	0.99 (0.15–1.83)	0.021	1.09 (0.10–2.09)	0.031

^a^Age per SD increase = 10 years.

^b^ *TP53* mutation burden per SD increase = 3.05E-5.

## Discussion

We have demonstrated that the combination of uterine lavage with ultradeep sequencing enables detection of ovarian cancer at two levels. First, tumor DNA was identified in more than two-thirds of lavages, even when present at very low frequency (<0.001), and from patients with early-stage cancers. Second, in addition to tumor cells, lavages also carried abundant *TP53* clonal expansions, which were more frequent and larger in patients with ovarian cancer. *TP53* MB, which captures both the number and size of clonal expansions, was associated with ovarian cancer independently of age and CA-125, suggesting potential as an ovarian cancer biomarker. Interestingly, while other ovarian cancer genes also showed extensive clonal expansions in lavage DNA, these expansions were observed at similar levels in patients with and without ovarian cancer, pointing to the unique role of *TP53* clonal expansions in association with ovarian cancer development.

The detection rate of *TP53* tumor driver mutations in lavages from patients with serous ovarian cancer was 79%, similar to the 80% detection rate that we reported in a prior study (11). For non-serous ovarian cancer, however, we detected the tumor mutation only in 33% of cases: 1/3 endometrioid carcinomas and 1/3 clear-cell carcinomas. While these numbers are small, they may reflect the different origins of disease. High-grade serous ovarian cancer often arises in fallopian tube epithelium, as can carcinosarcoma. However, clear-cell and endometrioid ovarian cancer generally arise in endometriosis. Therefore, uterine lavages might not capture cancer cells as frequently in cancers arising beyond the tubal lumen. Remarkably, the *TP53* driver mutation could be detected in five of six early-stage serous ovarian cancer cases indicating potential for the detection of early lesions. There are multiple potential explanations for the finding of tumor-specific *TP53* mutations in the uterine lavage, especially considering some cases involved cancer confined to an ovarian cyst. In some cases, tubal or myometrial involvement of the tumor can lead to dissemination of the *TP53-*mutant clones. Alternatively, cancer cells may travel through lymphatic or hematologic channels, or the captured clones may represent nonmalignant parallel somatic evolution. Furthermore, *TP53* foci (also known as *TP53* signatures) are common in fallopian tubes of women with HGSC or at high risk of HGSC (34) indicating that multiple *TP53-*mutant clones coexist and are potential precursors. *TP53*-mutant cells might exfoliate from these foci, in agreement with the precursor escape theory of HGSC carcinogenesis (35), and be collected in uterine lavage.

To clinically apply the detection of tumor-specific mutations for cancer diagnosis, tumor mutations must be distinguishable from background mutations without *a priori* knowledge of the tumor genetics. In our serous cohort, only 7 of 11 *TP53* driver mutations were present at VAF higher than 0.003 (all but one non-cancer case were below that threshold) and with more than 4× the VAF of the largest background mutation. While this sensitivity is limited for the identification of cancer cases without prior knowledge of the tumor mutation, we have discovered that *TP53* background mutations carry valuable information that could also be leveraged for ovarian cancer screening.

We found that uterine lavages detect multiple background mutations in cancer driver genes. More than half of these mutations were common cancer driver mutations, including canonical mutations in *KRAS* and *PIK3CA*. For two of the oncogenes (*KRAS* and *PIK3CA*) and three of the tumor suppressor genes (*PPP2R1A*, *ARID1A*, and *TP53*), cancer driver mutations were not only very abundant, but also had significantly higher VAF than non-driver mutations, indicating clonal expansion of mutant clones. On the basis of prior studies (36–39) and the nature of uterine lavage, it is likely that most of the mutations observed in lavage DNA originate in endometrium. Whole genome and target sequencing of endometrial glands have identified extensive clonal expansions in individuals without endometrial cancer, with more frequent somatic mutations seen in *KRAS* and *PIK3CA* specifically (36–38). In addition, a prior study using NGS of uterine lavage samples also identified somatic mutations in *PIK3CA*, *KRAS*, and *PTEN* (among other genes), representing clonal expansion in the normal endometrium (39). Overall, our results extend these prior findings by providing a high-resolution (VAF < 0.001) characterization of cancer driver mutations in uterine lavage of patients with and without ovarian cancer and provide strong evidence supporting the new paradigm of clonal evolution in normal tissue (19).

Interestingly, *TP53* was the only tested gene with a different frequency and burden of mutations in uterine lavages from patients with and without ovarian cancer. *TP53*-driven clonal expansions appeared to be linked to the development of ovarian cancer, with increased driver mutation VAF, mutational frequency, and mutational burden in ovarian cancer cases compared with benign cases. Notably, the high burden of *TP53* mutations in lavage was not always due to the specific tumor mutation alone but to other large *TP53*-mutant clones present in lavage DNA from serous as well as non-serous ovarian cancer. While we cannot determine the origin of the *TP53*-mutant clones in lavage, our data are consistent with the hypothesis that an increased burden of *TP53-*mutant clones is associated with the development of ovarian carcinoma. *TP53* clonal evolution is key to the current understanding of the pathogenesis of high-grade serous ovarian cancer as an evolutionary process initiated from *TP53-*mutant cells in the fallopian tube (40). Aside from the possibility of early precursor escape of *TP53-*mutant cells from *TP53* foci commonly found in fallopian tubes (34–35), excess of *TP53* clones observed in lavages from patients with serous ovarian cancer may correspond to endometrial *TP53* field effects extending to the fallopian tube epithelium. For non-serous cancers, the connection between *TP53* clonal expansions and cancer development is not clear because these cancers are driven by mutations in multiple genes other than *TP53* (21). The role of clonal expansions in carcinogenesis might not only be related to the direct growth of mutant cells but also to the generation of microenvironments that are permissive to the expansion of other mutant clones (19). Further studies with larger number of cases are warranted to explore this hypothesis. Nevertheless, from a clinical perspective, the measurement of *TP53* MB in uterine lavage appears as a promising tumor agnostic, minimally invasive molecular test for screening or risk stratification of ovarian cancer. This is an urgent need especially in the subset of patients at high-risk of ovarian cancer due to inherited *BRCA1* and *BRCA2* mutations (41). While cost considerations must be addressed when applying this tool to a larger population, feasibility in the outpatient setting has already been demonstrated (12).

Our study has two important limitations. First, it was based on convenience collection of gynecologic cases given the pilot study design, and therefore was not age matched. To address this limitation, we performed logistic regression including *TP53* MB, age and CA-125 as covariates and demonstrated that *TP53* MB was significantly associated with ovarian cancer in the model. Second, because the number of cases is small, we have no power to test associations and interactions with other ovarian cancer risk factors such as prior chemotherapy, endometriosis, or germline *BRCA* mutations. Future studies with larger sample sizes matched for age that allow for adjustment of ovarian cancer factors are currently under way.

In conclusion, by performing deep sequencing of uterine lavage with a panel of common ovarian cancer genes, we have demonstrated a prevalent process of clonal expansion in patients with and without ovarian cancer in agreement with multiple findings of clonal evolution in normal endometrium. While *TP53* tumor mutations can be found in lavages, the most relevant finding of our work is the discovery of increased *TP53* mutation burden in lavage DNA of patients with ovarian cancer. This exploratory work expands upon prior utilization of uterine lavage DNA for ovarian cancer detection (10, 11) by focusing on the associations between mutational burden and risk and using higher-fidelity methods to improve the accuracy and detection of low-frequency mutations. Although the sample size is small, these findings support the emerging notion that clonal expansions of certain cancer susceptibility genes, in this case *TP53*, might be linked to the development of cancer and may harbor clinical value as a biomarker for cancer risk thus informing future study design.

## Supplementary Material

Supplementary Methods Supplementary Methods

Supplementary Tables 1-6 Table S1. Clinical patient information
Table S2. Duplex Sequencing gene targets
Table S3. Custom probes for hotspots
Table S4. Mutation Frequency and Mutation Burden by patient and gene
Table S5. Codons most frequently mutated in ovarian cancers
Table S6. List of coding mutations by patient and gene

Supplementary Figures 1-11 S1. Experimental design
S2. Association between the variant allele frequency (VAF) of tumor mutations identified in uterine lavage with stage of the ovarian cancer (A) and preoperative CA-125 level (B)
S3. Gene distribution of the largest mutant clones identified in uterine lavage
S4. Correlations between Mutation Frequencies (MF) of genes commonly mutated in lavage DNA
S5. Comparison of Mutation Frequencies (MF) by patient group.
S6. Comparison of Mutation Frequency (MF) for individual genes in uterine lavages from patients with and without cancer
S7. Correlations between TP53 coding mutation frequency (MF) and age
S8. Distribution of driver and non-driver mutations identified in lavage DNA by gene and patient
S9. Mutation spectrum distribution of coding mutations detected in lavage DNA compared to ovarian cancer mutations reported in COSMIC
S10. Comparison of Variant Allele Frequency (VAF) of driver mutations in lavage DNA from patients with and without ovarian cancer
S11. Comparison of mutation burden (MB) of common ovarian cancer genes in lavage DNA from patients with and without ovarian cancer
