# Supplementary material for: Uterine Lavage Identifies Cancer Mutations and Increased TP53 Somatic Mutation Burden in Individuals with Ovarian Cancer
Source: Cancer Res Commun. 2022 Oct 27;2(10):1282–92. doi: 10.1158/2767-9764.CRC-22-0314 (PMC9615025; doi:10.1158/2767-9764.CRC-22-0314)
Supplement: Supplementary Methods [file crc-22-0314-s01.docx]

**SUPPLEMENTARY METHODS**

**Patients and samples**

The study included 34 women that underwent gynecological surgery for suspected masses at the University of Washington between November 2019 and October 2020. Routine pathological review of surgical specimens revealed that 14 patients had no pathology or benign histologies including cystadenoma, fibroma, and leiomyoma. The remaining patients had ovarian carcinomas including 1 with carcinosarcoma, 3 with clear-cell carcinoma, 3 with endometroid carcinomas, and 13 with high-grade serous ovarian carcinomas. The uterine pathology in cases in which a hysterectomy was performed was reviewed, without any cases of malignancy. Seven patients had a history of prior breast cancer, one of whom had chemotherapy, and one had prior melanoma. Two patients had endometriosis and four patients had prior tubal ligation. Tumors were surgically staged according to the International Federation of Obstetrics and Gynecology (FIGO) criteria. The BROCA sequencing panel was used to determine germline DNA mutations [1, 2]. Two ovarian cancer patients carried a germline *BRCA1* mutation (**Supplementary Table S1**).

**Duplex Sequencing analysis**

Sequencing reads were analyzed using pipeline v2.1.2 available at <https://github.com/Kennedy-Lab-UW/Duplex-Seq-Pipeline>. First, raw reads were grouped using the double stranded molecular tag included in the duplex adapters and a Single-Strand Consensus Sequence read was built from reads sharing the same tag. Then Single-Strand Consensus Sequence reads with complementary tags were compared to produce a single, highly accurate duplex read. Duplex reads were aligned to the human genome reference hg38 (GRCh38), end-trimmed, and overlap-trimmed.  Variants were called using VarDict Java [3], and output VCF files were converted to MAF files using the Vcf2Maf script (<https://github.com/mskcc/vcf2maf>) with VEP version 104 and gene transcripts as indicated in **Supplementary Table S2.** Gene transcripts corresponded to those with the most mutations recorded in COSMIC (v95) [4] and coincided with canonical transcripts in Ensambl, except for *BRAF*. Single Nucleotide Polymorphisms (SNP) were identified based on VAF (>0.3) and were used to confirm absence of cross-contamination. For each lavage, we quantified coding and non-coding mutations and the total number of coding and non-coding sequenced nucleotides. “Coding” included nucleotides in coding exons plus 2bp boundary nucleotides to capture splice site mutations, and “non-coding” included all the remaining nucleotides in the target regions. Masking was performed for exons that did not achieve a minimum depth of 250 in all samples (*PIK3CA* exon 10, *PPP2R1A* exon 2, and *PTEN* exons 2-3) and for *TP53* areas prone to sequencing artifacts (located in exons 4 and 11). Non-coding positions were required to be covered at >250x to be included for analysis.

R scripts were used to process MAF files to generate mutation frequencies and data plots. Coding mutations were extracted from MAF files and were further annotated by mutation type (missense, nonsense, splice, indel and synonymous), mutation spectrum (C>A, C>G, C>T, T>A, T>C and T>G), and cancer driver mutations (**Supplementary Table S6).**

**Tumor Duplex Sequencing**

For 8 high-grade serous tumors and all the non-serous tumors, DNA was extracted from formalin-fixed, paraffin-embedded (FFPE) tissue sections using QIAamp DSP DNA FFPE Tissue kits (Qiagen, Hilden, Germany). Tumor areas were previously identified in hematoxylin-eosin stained sections and were macrodissected prior to extraction. Between 20-100ng of tumor DNA were processed for library preparation using duplex sequencing kits (TwinStrand Biosciences). Non-serous tumors were captured with the same panel used for lavages and serous tumors were captured with a *TP53*-only panel. Libraries were sequenced using 151 PE reads on a MiSeq Illumina platform on site, allocating ~0.5 million clusters per sample. Raw reads were processed with the DS pipeline v2.1.2 available at <https://github.com/Kennedy-Lab-UW/Duplex-Seq-Pipeline>. A single clonal tumor mutation was identified in all cases except for the endometrioid tumor of patient P19, which carried 3 driver mutations in *ARID1A*, *CTNNB1* and *PIK3CA* (**Table 1**).

**References**

1. Walsh T, Casadei S, Lee MK, Pennil CC, Nord AS, Thornton AM, et al. Mutations in 12 genes for inherited ovarian, fallopian tube, and peritoneal carcinoma identified by massively parallel sequencing. Proc Natl Acad Sci U S A. 2011;108(44):18032-7.

2. Pennington KP, Walsh T, Harrell MI, Lee MK, Pennil CC, Rendi MH, et al. Germline and somatic mutations in homologous recombination genes predict platinum response and survival in ovarian, fallopian tube, and peritoneal carcinomas. Clin Cancer Res. 2014;20(3):764-75.

3. Lai Z, Markovets A, Ahdesmaki M, Chapman B, Hofmann O, McEwen R, Johnson J, Dougherty B, Barrett JC, and Dry JR. VarDict: a novel and versatile variant caller for next-generation sequencing in cancer research. Nucleic Acids Res. 2016; 20;44(11):e108.

4. Tate JG, Bamford S, Jubb HC, Sondka Z, Beare DM, Bindal N, et al. COSMIC: the Catalogue Of Somatic Mutations In Cancer. Nucleic Acids Res. 2019;47(D1):D941-D7.
