# Supplementary material for: Uterine Lavage Identifies Cancer Mutations and Increased TP53 Somatic Mutation Burden in Individuals with Ovarian Cancer
Source: Cancer Res Commun. 2022 Oct 27;2(10):1282–92. doi: 10.1158/2767-9764.CRC-22-0314 (PMC9615025; doi:10.1158/2767-9764.CRC-22-0314)

Supplementary Figures

Supplementary Figure S1. Experimental design

Supplementary Figure S2. Association between the variant allele frequency (VAF) of tumor mutations identified in uterine lavage with stage of the ovarian cancer (A) and preoperative CA-125 level (B)

Supplementary Figure S3. Gene distribution of the largest mutant clones identified in uterine lavage

Supplementary Figure S4. Correlations between Mutation Frequencies (MF) of genes commonly mutated in lavage DNA

Supplementary Figure S5. Comparison of Mutation Frequencies (MF) by patient group.

Supplementary Figure S6. Comparison of Mutation Frequency (MF) for individual genes in uterine lavages from patients with and without cancer

Supplementary Figure S7. Correlations between *TP53* coding mutation frequency (MF) and age

Supplementary Figure S8. Distribution of driver and non-driver mutations identified in lavage DNA by gene and patient

Supplementary Figure S9. Mutation spectrum distribution of coding mutations detected in lavage DNA compared to ovarian cancer mutations reported in COSMIC

Supplementary Fig S10. Comparison of Variant Allele Frequency (VAF) of driver mutations in lavage DNA from patients with and without ovarian cancer

Supplementary Figure S11. Comparison of mutation burden (MB) of common ovarian cancer genes in lavage DNA from patients with and without ovarian cancer

Supplementary Figure S1. Experimental design. A. Lavage of the uterine cavity at time of gynecologic surgery with 10ml of saline solution enables the collection of disseminated tumor cells (figure modified from Salk et al. [11]). **B.** DNA extracted from the lavage cell pellet underwent duplex sequencing targeting a panel of common ovarian cancer genes. **C.** Histology and FIGO stage of ovarian cancers of the 34 patients included in the study.

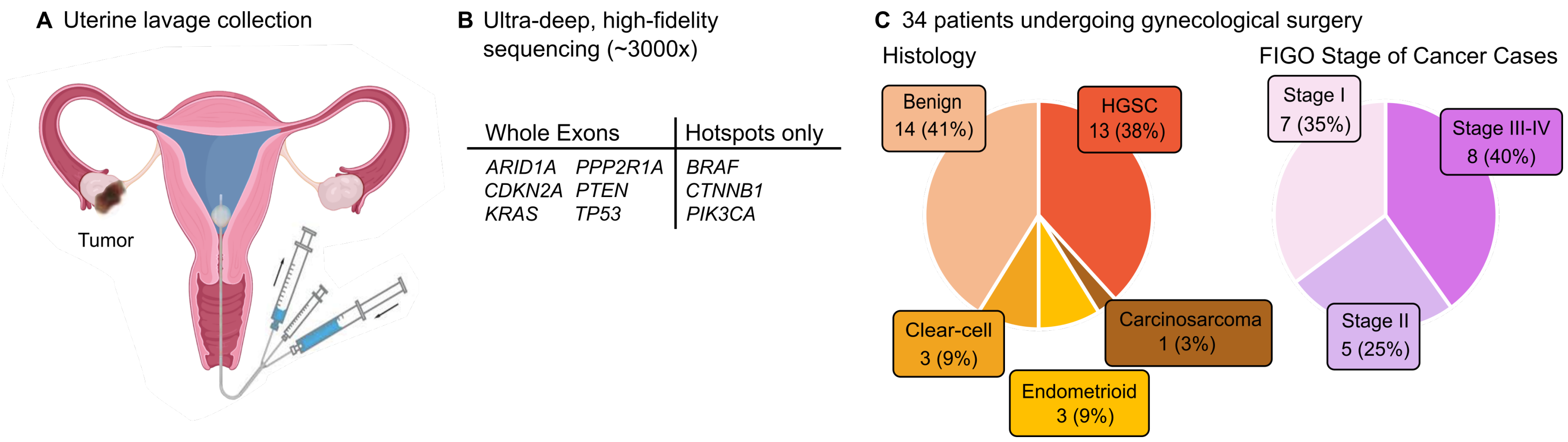

Supplementary Figure S2. Association between the variant allele frequency (VAF) of tumor mutations identified in uterine lavage with stage of the ovarian cancer (A) and preoperative CA-125 level (B). No significant associations were identified.

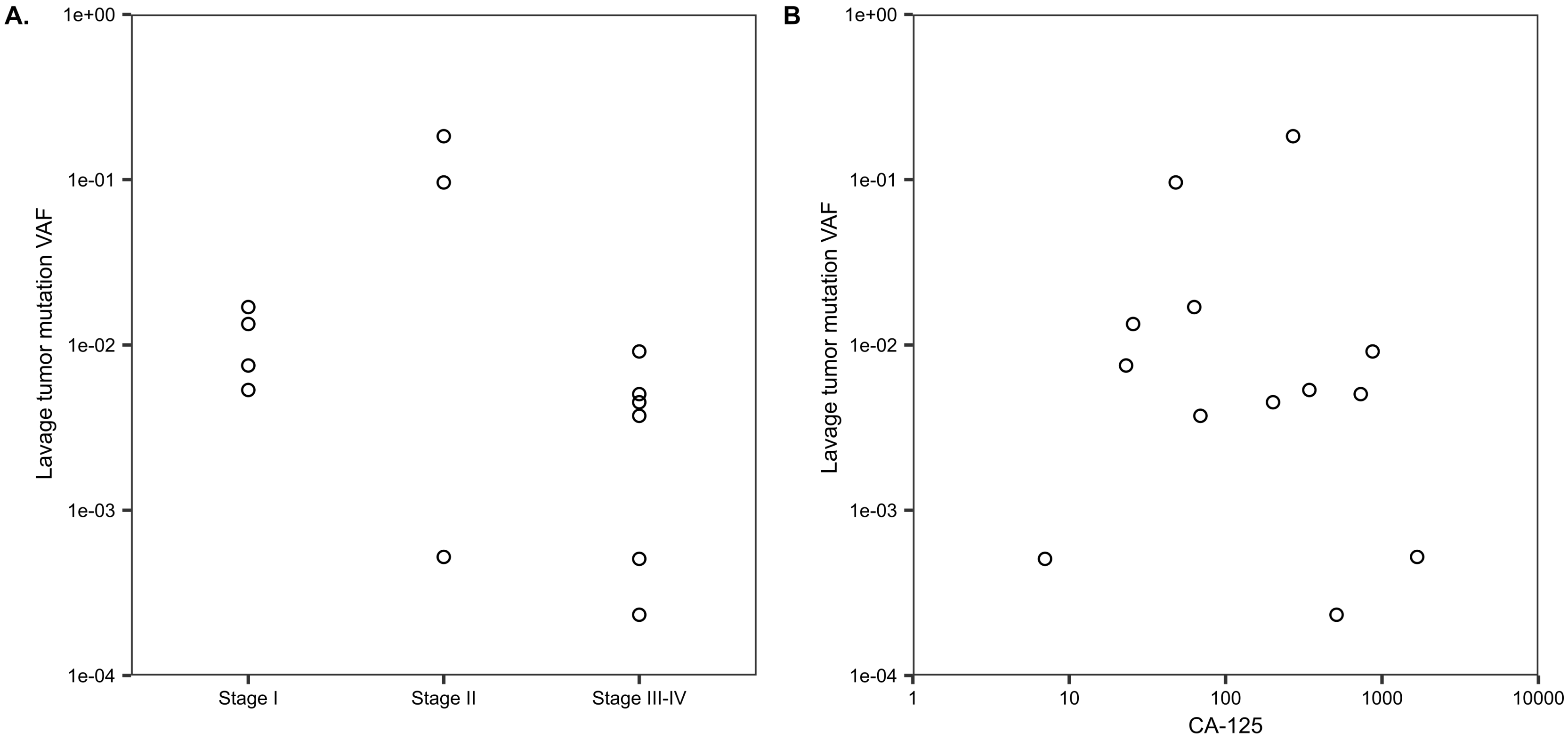

Supplementary Figure S3. Gene distribution of the largest mutant clones identified in uterine lavage. Somatic background mutations with variant allele frequency (VAF)>0.005 are plotted by descending VAF and color coded by gene. Tumor mutations are not included. Large somatic clones were identified in most genes sequenced.

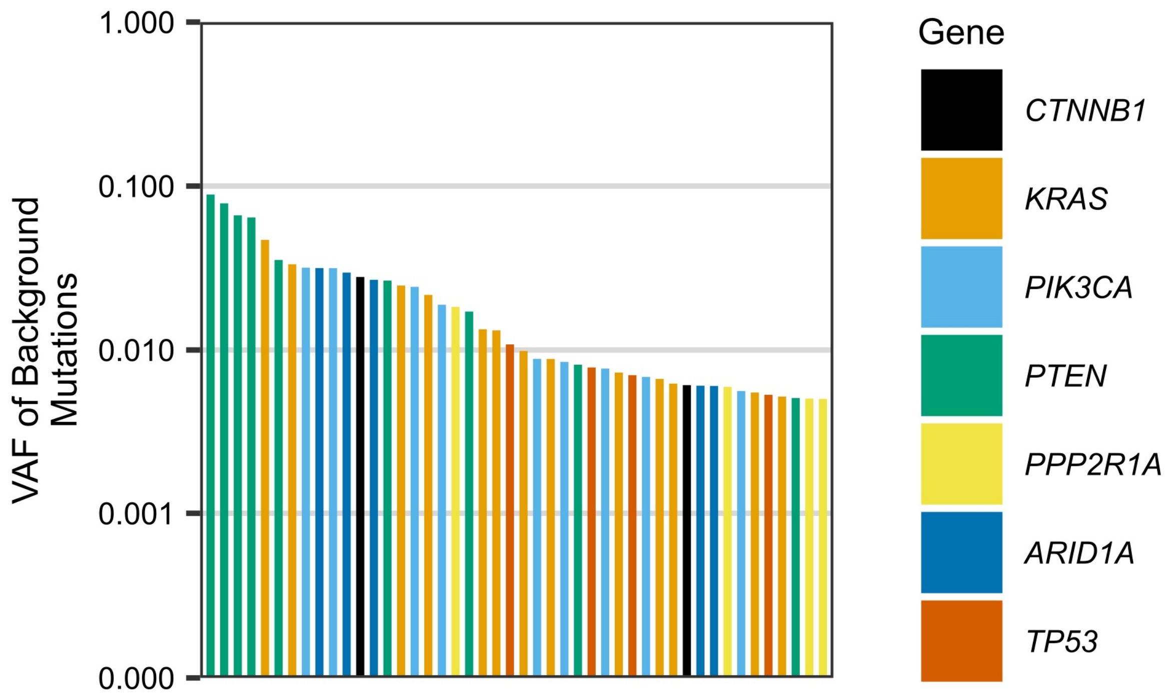

Supplementary Figure S4. Correlations between Mutation Frequencies (MF) of genes commonly mutated in lavage DNA. For each gene, MF is calculated as the number of unique coding mutations divided by the number of coding nucleotides sequenced. The table displays the rho coefficient for Spearman correlations with asterisks and color coding indicating the significance level. Correlations were calculated for genes that carried mutations in more than 50% of uterine lavage samples.

	ARID1A	*KRAS*	*PIK3CA*	*PPP2R1A*	*PTEN*	*TP53*
ARID1A	1.000	0.261	0.446**	0.331	0.408*	0.466**
KRAS		1.000	0.467**	0.587**	0.622**	0.489**
PIK3CA			1.000	0.557**	0.575**	0.437**
PPP2R1A				1.000	0.491**	0.375*
PTEN					1.000	0.355*
TP53						1.000

Significance level: Not significant; * p < 0.05; ** p < 0.005

Supplementary Figure S5. Comparison of mutation frequencies (MF) by patient group. MF is calculated by dividing the number of mutations detected by the number of nucleotides sequenced. Each circle represents the MF for an individual sample. p-values for Mann-Whitney U tests are shown for each comparison. The median MF for each group is indicated with a horizontal black bar. **A.** Comparison of coding and non-coding MF for all sequenced genes combined in uterine lavages from the 3 patient groups. **B.** Comparison of *TP53*-specific coding MF between patient groups.

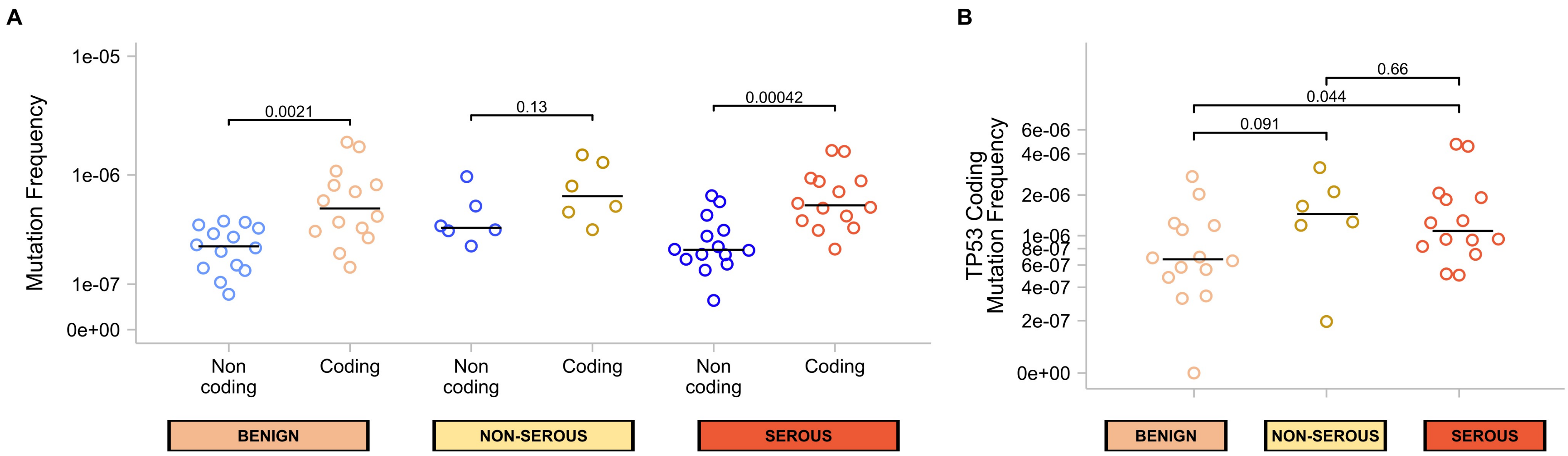

Supplementary Figure S6. Comparison of Mutation frequency (MF) for individual genes in uterine lavages from patients with and without cancer. MF is calculated as the number of unique coding mutations in a gene divided by the number of coding nucleotides sequenced**.** Only the genes that carried mutations in more than 50% of uterine lavages are shown, with the exception of *TP53*, which is shown in Fig. 3B. Horizontal bars indicate the median for each group and p-values correspond to Mann-Whitney U tests.

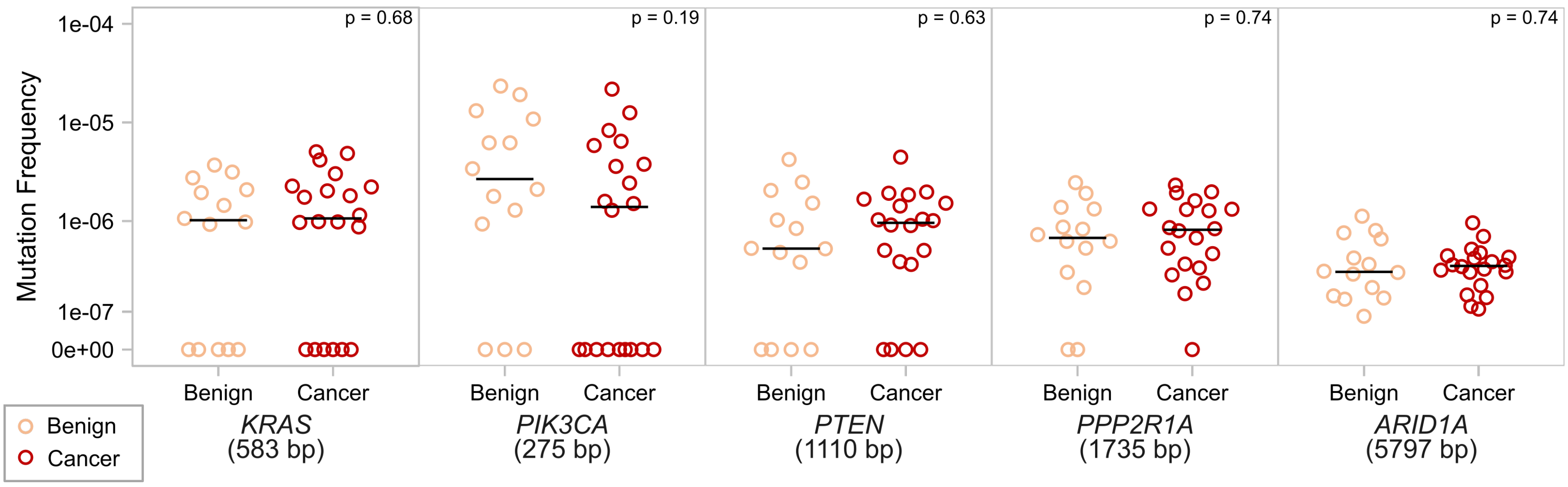

Supplementary Figure S7. Correlations between *TP53* coding mutation frequency (MF) and age. A. Correlation for the 34 patients included in the study. MF is calculated as the number of unique coding mutations in *TP53* divided by the number of coding nucleotides sequenced. **B.** Correlations separated by patient group based on diagnosis of ovarian cancer. Spearman correlation test ρ and p-value are displayed.

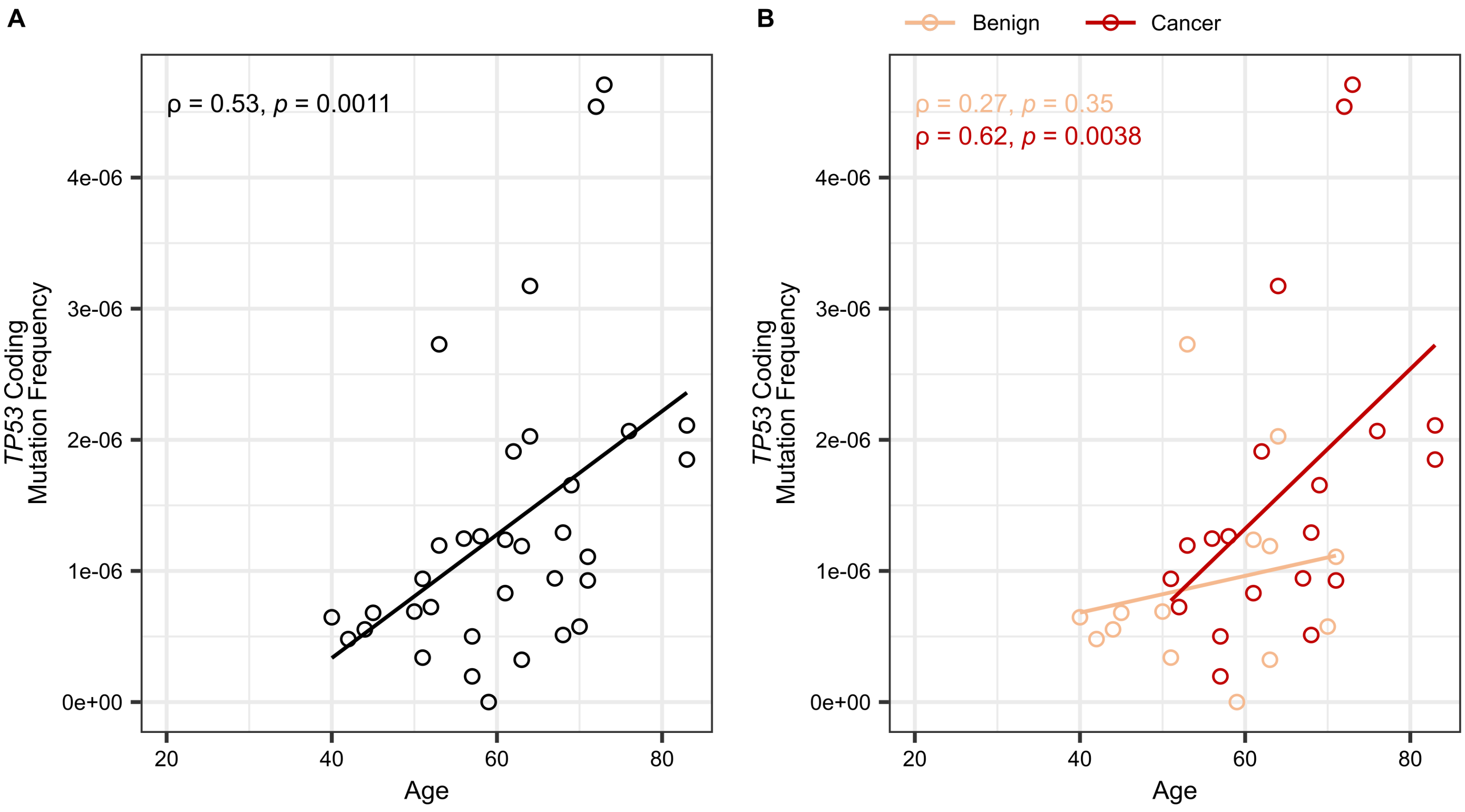

Supplementary Figure S8. Distribution of driver and non-driver mutations identified in lavage DNA by gene and patient. A. Counts of driver and non-driver mutations are indicated with color-coded bars for each patient and gene. Cancer driver mutations are defined, in oncogenes, as substitutions occurring in common hotspots codons and, in tumor suppressor genes, as substitutions occurring in common hotspots codons plus insertion/deletions, nonsense and splice mutations. **B.** For each gene, the proportion of driver vs. non-driver mutations are shown for patients with and without ovarian cancer. Proportions are color-coded with the actual number of mutations indicated for each category. Fisher exact test p-values are indicated to the right. NA: not applicable due to low number of mutations.

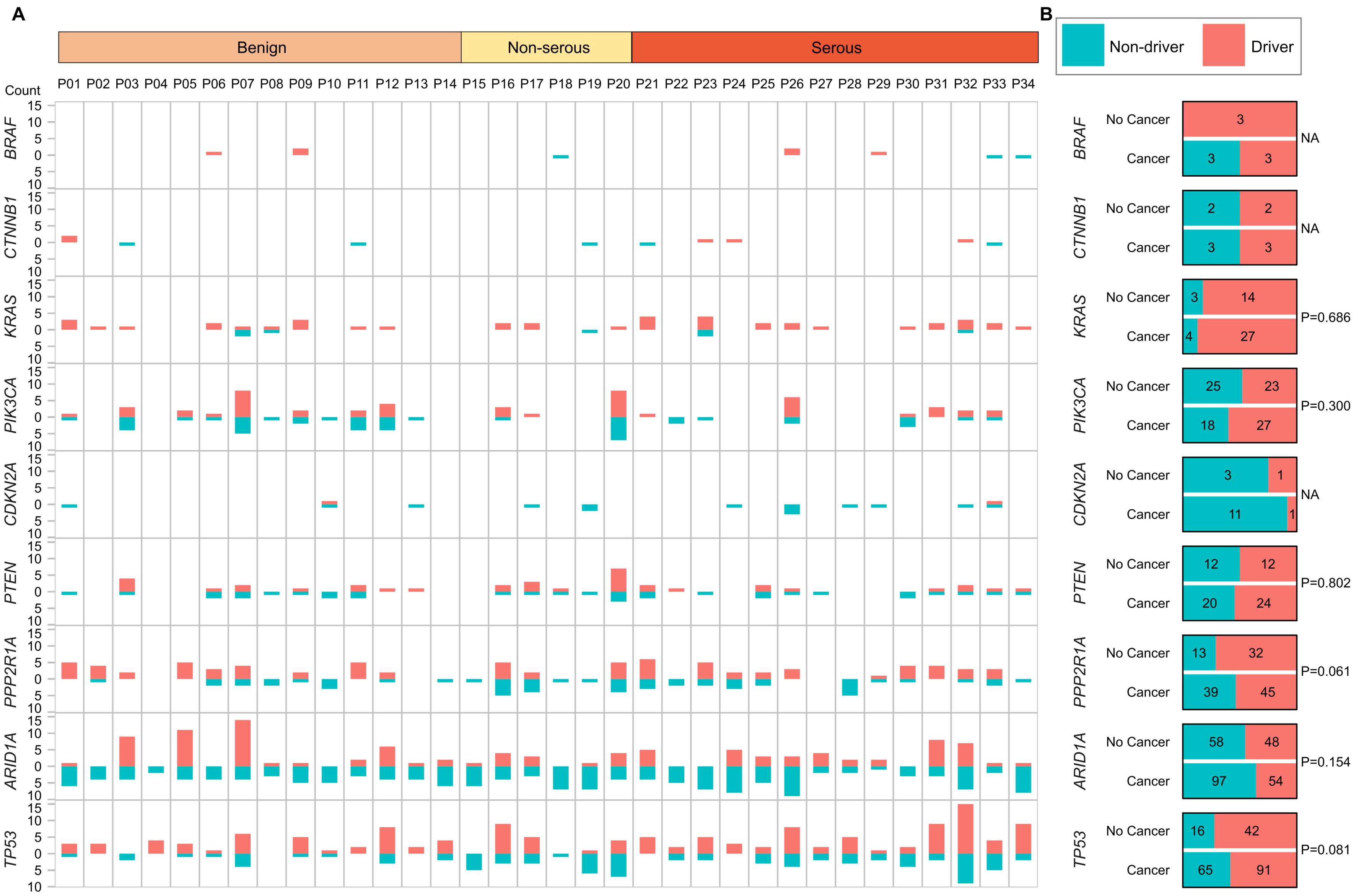

Supplementary Figure S9. Mutation spectrum distribution of coding mutations detected in lavage DNA compared to ovarian cancer mutations reported in COSMIC. Mutation distribution is showed along the y-axis color coded by substitution type. Mutations are divided by groups reflecting the age and pathology of the patients. Because changes in mutation spectrum are age-related, the 4 patients in the study younger than 50, who were all patients without ovarian cancer, are considered as a separate group. Patients with benign pathology that are older than 50 and all patients with non-serous or serous OC demonstrate a mutation spectrum in lavage DNA similar to that seen in ovarian cancer.

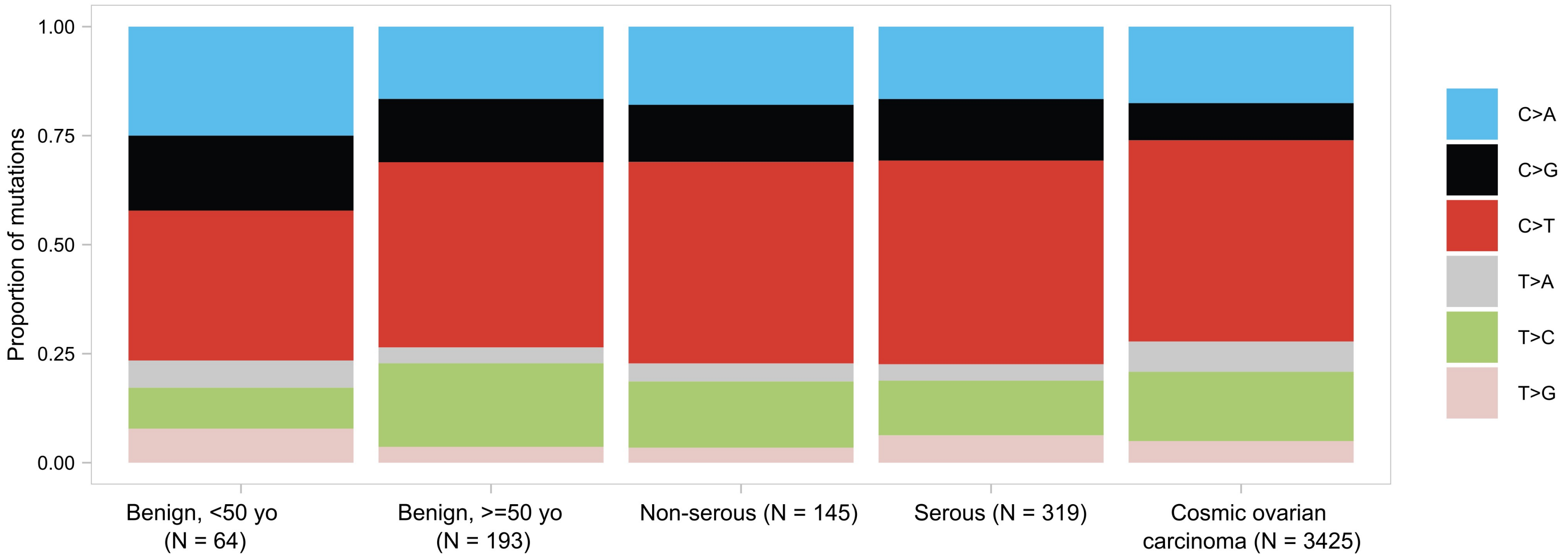

Supplementary Fig S10. Comparison of Variant Allele Frequency (VAF) of driver mutations in lavage DNA from patients with and without ovarian cancer. Cancer driver mutations are defined, in oncogenes, as substitutions occurring in common hotspots codons and, in tumor suppressor genes, as substitutions occurring in common hotspots codons plus insertion/deletions, nonsense and splice mutations. Only the genes that carried mutations in more than 50% of the uterine lavage samples are shown, with the exception of *TP53* which is shown in Fig. 5A. Each circle corresponds to a unique mutation. Overlying box plots display the quartiles with whiskers extending up to 1.5x the interquartile range. Bar plots above display the total number of mutations in each group. P-values correspond to Mann-Whitney U tests comparing the distribution of VAF of cancer driver mutations between benign and cancer patients.

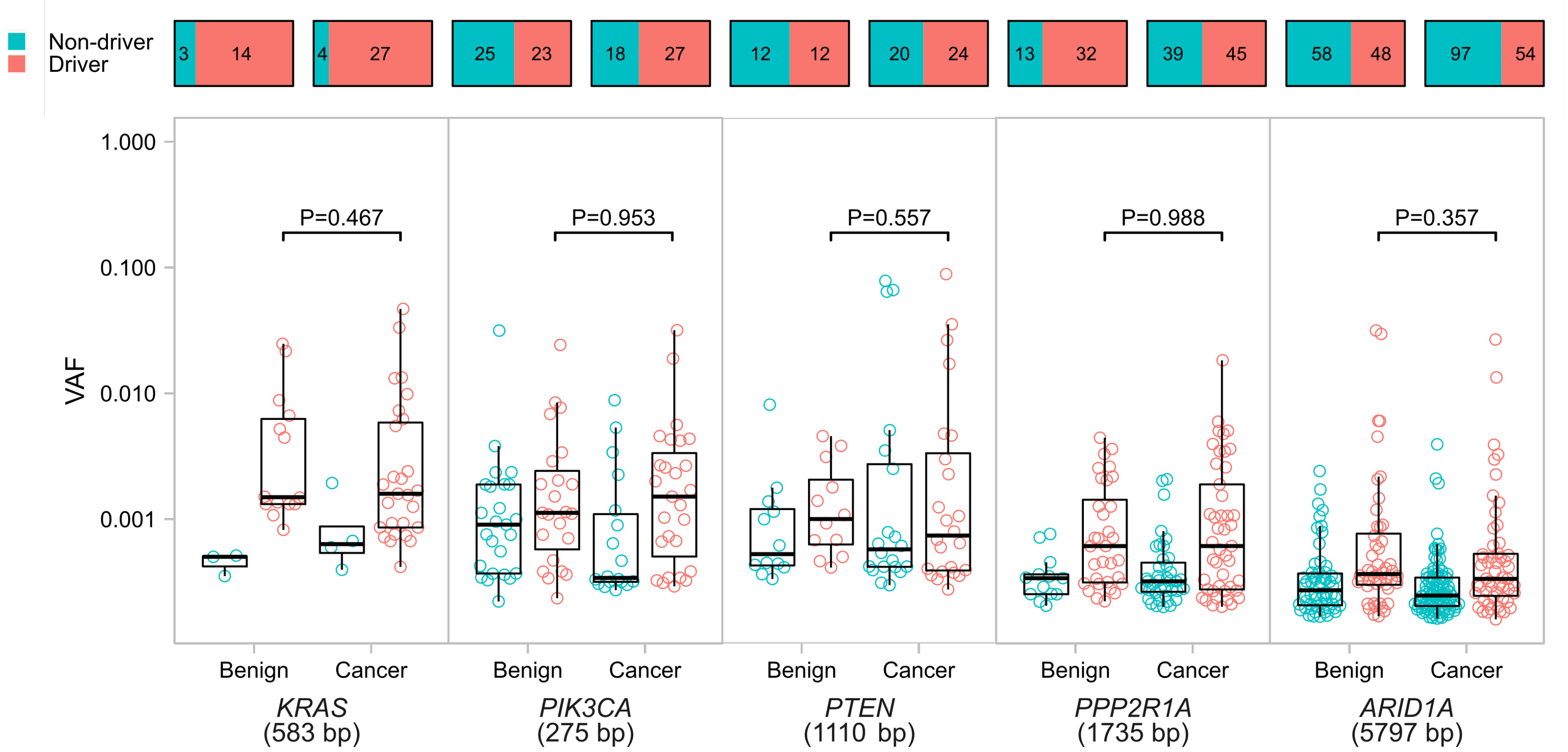

Supplementary Figure S11. Comparison of mutation burden (MB) of common ovarian cancer genes in lavage DNA from patients with and without ovarian cancer. For each gene, MB is calculated as the total number of mutant molecules identified in a lavage divided by the total number of nucleotides sequenced per gene. Only the genes that carried mutations in more than 50% of the uterine lavage samples are shown. Each circle indicates an individual lavage sample. Horizontal bars indicate the median for each group and p-values correspond to Mann-Whitney U tests.

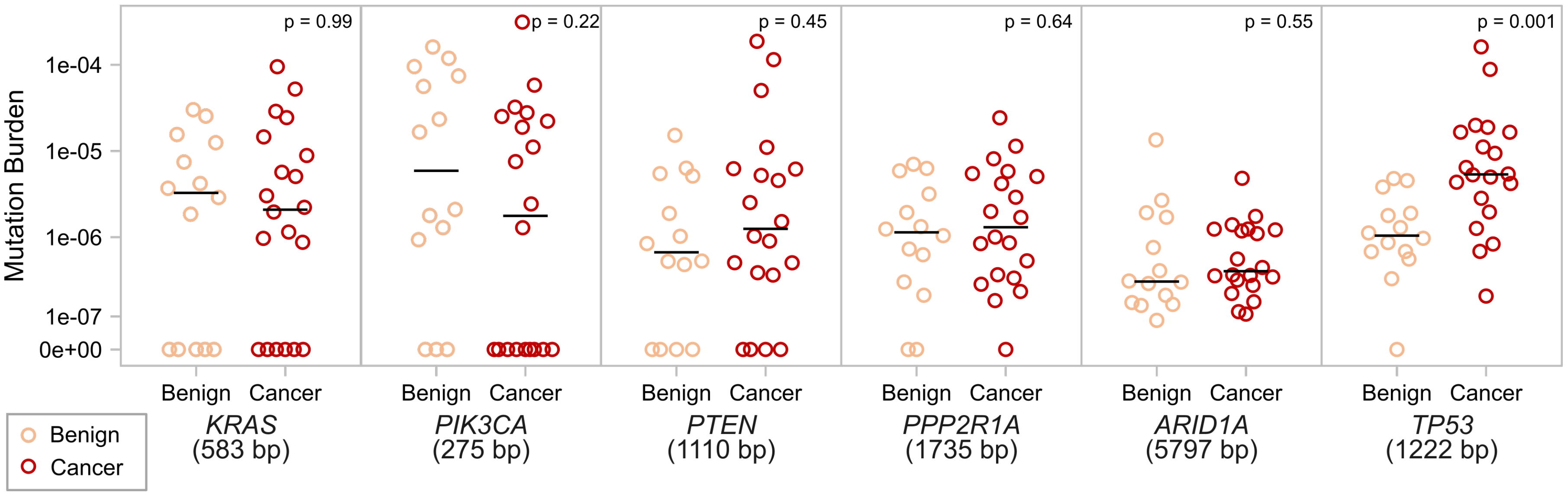

Supplement: Supplementary Figures 1-11 — S1. Experimental design S2. Association between the variant allele frequency (VAF) of tumor mutations identified in uterine lavage with stage of the ovarian cancer (A) and preoperative CA-125 level (B) S3. Gene distribution of the largest mutant clones identified in uterine lavage S4. Correlations between Mutation Frequencies (MF) of genes commonly mutated in lavage DNA S5. Comparison of Mutation Frequencies (MF) by patient group. S6. Comparison of Mutation Frequency (MF) for individual genes in uterine lavages from patients with and without cancer S7. Correlations between TP53 coding mutation frequency (MF) and age S8. Distribution of driver and non-driver mutations identified in lavage DNA by gene and patient S9. Mutation spectrum distribution of coding mutations detected in lavage DNA compared to ovarian cancer mutations reported in COSMIC S10. Comparison of Variant Allele Frequency (VAF) of driver mutations in lavage DNA from patients with and without ovarian cancer S11. Comparison of mutation burden (MB) of common ovarian cancer genes in lavage DNA from patients with and without ovarian cancer [file crc-22-0314-s03.pdf]
